# Molecular Profiling–Based Precision Medicine in Cancer: A Review of Current Evidence and Challenges

**DOI:** 10.3389/fonc.2020.532403

**Published:** 2020-10-27

**Authors:** Qi Zhang, Qihan Fu, Xueli Bai, Tingbo Liang

**Affiliations:** ^1^ Department of Hepatobiliary and Pancreatic Surgery, The First Affiliated Hospital, Zhejiang University School of Medicine, Hangzhou, China; ^2^ The Key Laboratory of Pancreatic Diseases of Zhejiang Province, Hangzhou, China; ^3^ The Innovation Center for the Study of Pancreatic Diseases of Zhejiang Province, Hangzhou, China; ^4^ Zhejiang Clinical Research Center of Hepatobiliary and Pancreatic Diseases, Hangzhou, China; ^5^ Department of Medical Oncology, The First Affiliated Hospital, Zhejiang University School of Medicine, Hangzhou, China

**Keywords:** precision medicine, molecular profiling, matched therapy, clinical trial, next-generation sequencing

## Abstract

Matched therapy based on next-generation sequencing is now a part of routine care to guide the treatment of patients with advanced solid tumors. However, whether and to what extent patients can benefit from this strategy on a large scale remains uncertain. In the past decade, several clinical studies were performed in this field, among which only one was a randomized trial. We reviewed the literature on this topic and summarize the existing data about the efficacy of this treatment strategy. Currently, the evidence is promising but not solid. Multiple ongoing trials are also summarized. We also discuss the limitations of this treatment strategy and certain unsolved important problems, including how to select the sample and target level, how to interpret the results, and the problem of drug accessibility. All these issues should receive more attention in future clinical trial design and the application of target therapy in cancer treatment.

## Introduction

Cancer control has now become more and more challenging in human society because of its increasing incidence (both current and predicted). Thanks to the advances in molecular medical research, strategies for cancer treatment are evolving from traditional therapies, such as surgery, chemotherapy, and radiotherapy, toward targeted therapy. Recently, precision medicine has been proposed as the future of cancer treatment, providing a revolutionary understanding of cancers according to their genetic alterations instead of their primary locations. It is believed that by targeting these “driver” genetic alterations or oncoproteins, clinicians could hit the “Achilles heel” of cancers.

Cancer cells may have somatic genetic alterations leading to abnormal expression of mRNA and proteins, allowing them to escape the usual controls on cellular growth. Knowledge of the genomic landscape might help to guide the diagnosis and therapy of multiple types of cancers. Genetic alterations in EGFR, ALK, ROS1, MET, HER2, KIT, BRAF, and germline BRCA1/BRCA2 have been shown to confer survival benefits on patients with certain solid tumors, including non-small cell lung cancer (1, 2), breast cancer (3), and melanoma (4). Certain tumors with different tissues of origin have been found to be similar at the molecular level, and it would seem reasonable to treat them using the same strategy (5). Molecular alternations, such as BRAF mutations (6), NTRK family fusion (7), and microsatellite instability (8), have been linked to responses to matched agents in a variety of tumor types. Many basket trials (a clinical trial in which patient eligibility relies on the presence of a specific genomic alteration without taking into account their histology) further confirm that targeted drugs might work without regard to tumor histology (9). Genome-driven cancer treatment is, thus, a promising strategy, and in the near future, tumor genetic testing will be part of the standard management of many cancers.

More and more types of cancer have been genetically profiled (10–16), and it is attractive for both physicians and investigators to make a treatment decision for a patient simply by testing the genetic mutations or oncoproteins. However, various concerns exist before such oncotarget-based therapy can be widely accepted. For instance, the majority of genomic alterations are biologically insignificant for cancer cell survival, and recognition of treatment-meaningful genomic alterations is critical but also mostly intractable (17, 18). In the market and even the laboratory, only a few drugs or chemicals are available for molecular profiling–based therapy. In addition, the companion diagnostics to detect targets are complicated and require confirmation using prospective clinical trials. Although several pilot studies using traditional molecular profiling methods, such as polymerase chain reaction (PCR), immunohistochemistry (IHC), fluorescent *in situ* hybridization (FISH), and microarrays demonstrate that patients could benefit from targeted therapy (19, 20), these methods have limited coverage of oncogenes and oncoproteins, and thus, the prognostic benefits of molecular profiling–based therapy have been underestimated.

The next-generation sequencing (NGS) technologies permit an unbiased analysis of cancer genomes. NGS has enabled the rapid detection of thousands of cancer-related genes using small quantities of DNA. Recent advances in timeliness and cost have made NGS financially available in academic cancer centers and commercial testing laboratories. The mutational landscape of metastatic cancer revealed from prospective clinical sequencing of 10,000 patients shows that about 40%–80% of patients subjected to NGS testing had more than one molecular alteration, and the median number of mutations per patient was five. TP53, KRAS, and PIK3CA are the most frequently mutated genes (21). Disappointingly, although 40% of the patients had potentially actionable alterations, less than 25% of them could be ultimately treated using suitable drugs (22–25). Many patients were unable to receive matched therapies because of poor-quality biopsies, lack of clinical trials or off-label use of drugs, and poor clinical state and performance status.

## Current Evidence of Proof of Concept for Molecular Profiling–Based Therapy

An early study from the Princess Margaret Hospital shows that four of six patients responded to matched therapy (26). In a cohort at the Dartmouth-Hitchcock Medical Center, two out of four patients experienced clinical benefit lasting more than 10 months (27). In a cohort at Johns Hopkins Medicine, 11 patients were treated with off-label target drugs, and 13 patients were enrolled in clinical trials with matched therapies; among them, the median progression-free survival was 5 months (28). Another pilot study from Korea proposed targeted therapy based on NGS for patients with refractory solid tumors. Although only 3 of 25 patients finally received targeted therapy, all of them experienced a partial response (29). Inspired by these pioneer studies, the large, prospective, single-arm study MOSCATO-01 (NCT01566019) trial was conducted, and the results are also encouraging (30). In this cohort, 199 of 1035 heavily treated patients were subject to targeted therapies, and their median overall survival (OS) was 11.9 months. This study compared the progression-free survival (PFS) of matched therapy (PFS2) with the PFS of the most recent therapy (PFS1) and found the PFS2:PFS1 ratio was >1.3 in 33% of the patients. A PFS2:PFS1 >1.3 indicates a treatment benefit given that PFS decreases over the lines of therapy in the natural course of the disease. Another large prospective trial (ProfiLER) shows 163 patients were assigned to matched therapy, and 23 (14.3%) patients had an objective response (22). Despite their single-arm design, these case series and trials suggest that NGS-based matched therapy might be promising in future cancer management.

Several large prospective studies attempted to find out whether targeted drugs matched with tumor molecular alterations are superior to conventional unmatched therapy. A retrospective study analyzed the outcome for 36 patients who received NGS-based genomic testing and targeted therapy. The average PFS was 22.9 weeks, and the PFS in the control group was only 12 weeks (31). In an early trial including 407 patients, the 103 patients who received molecular targeted agents showed a significantly higher response rate compared with those in the non-matched treatment group (42.6% vs. 24.3%, *P* = 0.009) (32). Another observational cohort enrolling 347 patients with advanced solid tumors also reports encourage results. Although significantly fewer patients in the matched therapy group were treated as first-line therapy, they had better PFS compared with those who did not receive matched therapy (33). The results of these trials are encouraging; however, none of them reports improved OS by matched therapy.

Results of further studies have been released in recent years and confirm that therapy matched to genomic variants is associated with improved OS. Investigators from the MD Anderson Cancer Center performed the large-volume IMPACT trial across tumor types, which was started in 2007. Compared with patients treated with unmatched therapy, those treated with matched therapy had a higher response rate (11% vs. 5%, *P* = 0.0099), longer PFS (3.4 vs. 2.9 months, *P* = 0.0015), and longer OS (8.4 vs. 7.3 months, *P* = 0.041) (34). Notably, in the matched group, the responders had significant longer OS compared with that of nonresponders (23.6 vs. 8.4 months, *P* < 0.001), and no difference was observed in the unmatched group. The Know Your Tumor Registry Trial focused on pancreatic cancer, a tumor type that has limited standard targeted therapy choices. In it, 26% patients had actionable molecular alteration, which was inconsistent with other types of cancer. The 46 patients who received a matched therapy had the better OS (2.58 vs. 1.51 years, *p* = 0.0004) compared with those who had unmatched therapy (35).

These data support the use of matched therapy when actionable gene mutations are detected. However, the investigation was observational and nonrandomized, and the outcome could be influenced by unknown biases. For example, the majority of the patients were assigned to nonmatched therapy because of a failure to detect any actionable molecular alterations. Hence, the groups of matched therapy and conventional therapy might have a difference in genomic background, which could affect the outcome.

The SHIVA trial (NCT01771458) was the first and also the only completed up-to-date randomized large-scale basket trial in this field (36). Patients with molecular alterations of three cancer-related signaling pathways (i.e., hormone receptor, PI3K/AKT/mTOR, and RAF/MEK pathways) were randomized to one group using matched molecularly targeted agents (experimental group, *n*=99) and another group receiving treatment according to the physicians’ choice (control group, *n*=96) (36). The median PFS was 2.3 months in the experimental group and 2.0 months in the control group. The difference was not statistically significant. Given that the patients enrolled were heavily treated, the potential of benefit from matched treatment was small, which might not be able to be detected because of insufficient power.

In order to figure out the subgroup patients who can benefit more from matched therapy, the concept of a matching score was set up. In another prospective, single-center study, the investigators defined the matching score as the number of matched agents over the number of gene alterations present and found that a high matching score was independently and significantly associated with better outcomes (37). They also found that patients with direct matches had a longer time to failure (TTF) and OS, and disease control [defined as stable disease (SD) ≥6 months, partial response (PR), or compete response (CR)] rates were higher in the indirectly matched patients. The newly published I-PREDICT trial, which included 149 consented and 83 treated patients with metastatic cancers, shows that up to 30% of patients evaluable for response achieved disease control (defined as SD ≥ 6 months) (38) and that a similarly high matching score was an independent factor of favored outcomes.

## Ongoing Trials of Molecular Profiling–Based Therapy

Following the encouraging results from previous trials, many large-volume trials are currently ongoing (39) (**Tables 1** and **2**). Most of these trials are designed as basket trials and have enrolled patients with various cancer types that share the same genetic abnormality. The U.S. National Cancer Institute (NCI) launched the NCI-MPACT (NCT01827384) trial in 2013 and the NCI-MATCH (NCT02465060) trial in 2015. All patients enrolled in these trials were subjected to repeated biopsy before therapy to obtain tumor specimens. The NCI-MPACT trial recruited patients with advanced cancer and assigned them to four treatment arms according to their molecular aberrations. This trial only performs DNA sequencing for 380 unique actionable variants in 20 genes that can be targeted by their four predefined treatments. The NCI-MATCH trial expanded the design of NCI-MPACT and contains up to 25 targeted treatments across all cancer types. The tumor specimens undergo both DNA and RNA sequencing to identify 143 genetic abnormalities. More than 6400 patients have already been enrolled in the NCI-MATCH trial, which is estimated to end in 2022 (43). Although NCI-MATCH is not a randomized trial, it is considered to be the largest precision medicine cancer trial based on the number of patients, treatment options, and types of cancers.

**Table 1:** Current evidence for molecular profiling–based therapy.

	Study period	Country	Sample	Seq Method	Patients	≥1 alteration	Actionable mutations	MT	Non-MT	Outcome
IMPACT (34)	2012-2013	USA	Tumor DNA	PCR-base seqIHC/NGS	1436	1179 (82.1%)	914 (63.7%)	390	247	ORR: 11% vs. 5% PFS: 3.4 vs. 2.9 months OS: 8.4 vs. 7.3 months
MOSCATO-01 (30)	2011-2016	USA	Tumor DNATumor RNA	aCGH/NGS//RNA-Seq/IHC/FISH	1035	/	411(40%)	199 (19%)	/	ORR: 11%PFS1/PFS2 ≥ 1.3: 63(33%)OS: 11.9 months
SHIVA (36)	2012-2014	France	DNA	NGSIHC	741	/	293 (40%)	99	96	PFS: 2.3 vs. 2.0 months
PREDICT-UCSD (33)	2012	USA	Tumor DNA	NGS	347	/	/	87 (25%)	93 (26.8%)	SD≥6 months/PR: 34.5 vs. 16.1%PFS: 4.0 vs. 3.0 monthsPFS2/PFS1 ≥ 1.3: 45.3 vs. 19.3%
	2014-2015	USA	ctDNA	NGS	168	96 (58%)	74(44%)	33 (19%)	39 (23.2)	SD≥6 months/PR: 42 vs. 7.1%
ProfiLER (22)	2013-2017	France	Tumor DNA	aCGH/NGS	2579	1032	699 (27%)	163	/	ORR: 13%
NEXT-1 (32)	2013-2014	Korea	DNA	NGS	428	342 (80%)	106 (25%)	103 (24%)	226 (53%)	42.6% vs. 24.3%
WINTHER (40)	2013-2015	USA	Tumor DNATumor RNA	NGS	303	/	/	107	/	SD≥6 months/PR/CR: 26.2%PFS1/PFS2 ratio ≥ 1.5:22.4%
CoPPO (41)	2013-2017	Denmark	Tumor DNATumor RNA	NGS	591	/	352 (70%)	101(20%)	/	ORR: 15%PFS: 3months
MD Anderson Cancer Center (34)	2014	USA	Tumor DNA	NGS	500	322 (64.4%)	/	122(24%)	66(13%)	PFS: 2.8 vs. 2.1%
I-PREDICT (38)	2015-2017	USA	Tumor NDActDNA	NGS/IHC	149	/	/	73	9^#^+1	SD≥6 months: 30%PFS: 3.67 vs. 1.93 monthsOS: 11.8 vs. NR
TARGET (42)	2017-2018	UK	ctDNA	NGS	100	/	41(41%)	11(11%)	17 (17%)^#^	ORR: 36.3% vs. 0%

aCGH, a comparative genomic hybridization analysis; MT, matched therapy; Non-MT, unmatched therapy; IHC, immunohistochemistry; FISH, fluorescent in situ hybridization; NGS, next generation sequencing; ORR, objective response rate; PFS, progression-free survival; ctDNA, circulating tumor DNA; SD, stable disease; RCT, randomized controlled trial; OS, overall survival; PFS2, PFS of matched therapy; PFS1, PFS of the most recent therapy.

^#^patients with actionable mutations received unmatched therapy.

**Table 2:** Ongoing Trials.

	Study period	Country	Disease	Study design	Seq sample	Method	Treatment	Endpoint
NCI-MPACTNCT01827384	2013-2020	USA	Advanced malignant solid tumor	Basket	Tumor DNA	NGS	Target Therapy(4 treatment arms)	ORRPFS
NCI-MATCHNCT02465060	2015-2022	USA	Advanced malignant solid tumorLymphomaMultiple myeloma	Basket	Tumor DNARNA	NGS	Target Therapy(25 treatment arms)	ORR6-months PFS
TAPURNCT02693535	2016-2021	USA	Solid tumorLymphomaMultiple myeloma	Basket	Tumor DNActDNATumor Protein	Genetic testIHC	Target Therapy(17 treatment arms)	ORRSD ≥16 weeks
HETIAN64NCT03239015	2017-2020	China	Solid Tumor	Basket	Tumor DNA	NGS	Target Therapy(11 treatment arms)	ORR
IMPACT2NCT02152254	2014-2020	USA	Advanced malignant solid tumor	RCT	Tumor DNA	NGS	Target Therapy vs Standard-of-Care treatment	PFS
CUPISCONCT03498521	2018-2022	USA	Cancer of unknown primary site	RCT	Tumor DNA	NGS	Target Therapy vs Chemotherapy	PFSOSORR

NGS, the next generation sequencing; ORR, objective response rate; PFS, progression-free survival; ctDNA, circulating tumor DNA; SD, stable disease; RCT, randomized controlled trial; OS, overall survival.

The American Society of Clinical Oncology (ASCO) initiated a clinical trial named Targeted Agent and Profiling Utilization Registry (TAPUR; NCT02693535). The TAPUR trial is a phase II, prospective, nonrandomized, multibasket trial that aims to assess the efficacy of targeted anticancer therapies. In addition to solid tumors, non-Hodgkin lymphoma and multiple myeloma could be included. There are 17 treatment arms in the TAPUR trial according to the molecular alterations, and all the drugs used are commercially available. To define the molecular alteration, genetic testing of tumor DNA and circulating tumor DNA (ctDNA) and IHC testing of tumor protein expression are all acceptable (44). Another phase II study, called the HETIAN64 trial (NCT03239015), was launched in 2016, which is the first NGS-based basket trial in China. In this trial, individuals with all types of solid tumors are recruited. If actionable molecular alterations are found *via* NGS testing, patients could be assigned to 11 treatment arms accordingly.

The IMPACT2 trial (NCT02152254) was initiated in 2014 by the MD Anderson Cancer Center and comprises a randomized prospective study comparing targeted therapy with standard-of-care therapy in metastatic cancer. The molecular profiling includes not only genomic profiling, but also immune markers, tumor mutational burden, microsatellite instability status, and/or transcriptomic analysis. The treatment options could be single agents or combinations. Another ongoing randomized controlled trial is the CUPISCO (NCT03498521) trial, which began in 2018 and aims to compare targeted therapy with platinum-based chemotherapy in patients with cancer of unknown primary site. In this trial, immunotherapy with Atezolizumab is included and is regarded as genomic alteration–based targeted therapy. Another two ongoing clinical studies are exploring the question of whether patients could gain addition benefit from using a broader panel (NCT03163732) or whole exome sequencing (NCT01774409). The results of these clinical trials could accelerate the implementation of precision oncology and guide the better use of targeted drugs. More studies, especially randomized clinical trials, are warranted in the field of precision medicine to prove the rationale of precision medicine in cancers (45).

## Unsolved Questions and Future Direction

**Figure 1:**
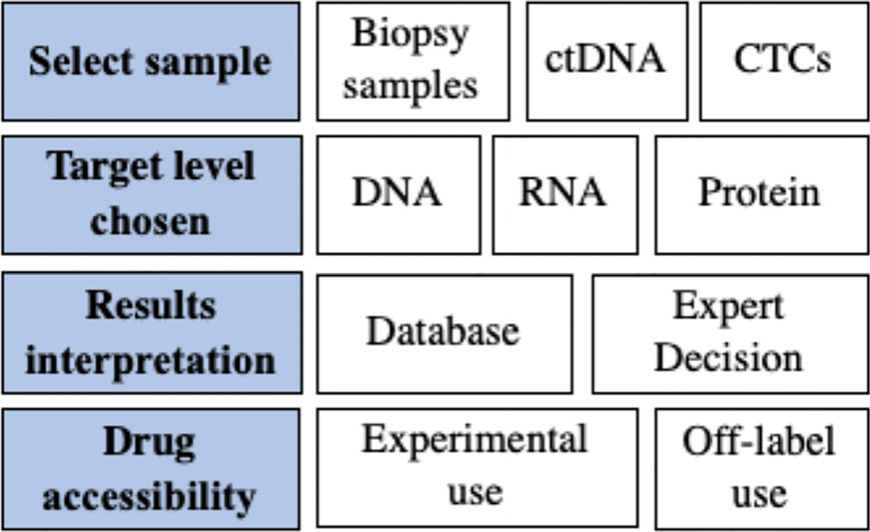
Summary of unsolved questions and future directions.

Precision medicine uses -omics technologies and platforms to profile genomic/transcriptomic/proteomic aberrations of cancers, aiming to guide treatment decision making by predicting the tumor’s response to matched agents. The concepts and rationales of precision medicine are clear; however, solid evidence of clinical benefits is challenging. Current evidence shows promising results; however, the quality of the evidence is a concern because of difficulties in the study design. Additionally, unlike classic clinical trials, comparisons of various precision medicine trials are difficult because many parameters of the trials are trial-specific. The unsolved questions and challenges are summarized in **Figure 1**.

### Origin of the Samples for Molecular Profiling

In the majority of trials, archival or fresh biopsy samples are the main source of materials used for molecular profiling. However, molecular profiling in up to 30% of patients failed because of a lack of archived tissue or poor sample quality (21). In addition, archival samples are unable to reflect information concerning tumor evolution and raise concerns related to their representativeness because of significant intratumoral heterogeneity (46, 47). Indeed, tumor heterogeneity is one of the biggest obstacles to improving the therapeutic response rate. Tumor heterogeneity is complex and changes dynamically, implying that none of the patients have the same disease. Each patient has tumor lesions with significantly distinct genetic landscapes that evolve over time; thus, analysis of a single lesion at a specific time provides very limited information for clinical decision making (48). It is, thus, difficult and impractical to capture the comprehensive genomic landscape of a patient’s cancer accurately. Given that performing multiple and repeated tissue biopsies is not feasible in clinical practice, liquid biopsy is proposed as an alternative method.

DNA fragments that are released from both primary and metastatic lesions are termed ctDNA, which is believed to provide a landscape of tumor genomic alterations (49) and suggests that ctDNA is an alternative or even better material for molecular profiling of cancers. The ctDNA from most patients with cancers displays at least one detectable altered gene, of which TP53, EGFR, KRAS, and PIK3CA are the most frequently detected (50). Sequencing of ctDNA reveals high concordance with tumor biopsies for the identification of critical driver mutations (51, 52); however, other studies report that ctDNA provides more general genomic alterations of cancers and has low concordance with the results from samples of primary or metastatic lesions (53, 54). Given the advantage of minimal invasion as a procedure and the capability of dynamic monitoring of tumor genomic evolution, the role of ctDNA in precision medicine has been increasingly explored.

A pilot small-volume study shows that 42% of patients matched to treatment that targeted alterations detected using ctDNA sequencing achieved SD >6 months or a PR, compared with 7.1% of the unmatched patients (55). The TARGET trial was launched to determine whether ctDNA sequencing was helpful for clinical decision making. For the first 100 enrolled patients, a 641-gene ctDNA NGS panel was successfully used in 99 of them, among whom 41 had actionable mutations. Eleven of the 41 patients received matched therapy, and 17 of them received unmatched therapy (42). The response rate in the match therapy group was 36%, and the median duration of therapy was 6 months. In comparison, none of the patients receiving unmatched therapy recorded a tumor response. These studies confirmed that noninvasive detection of actionable alterations from ctDNA could be technically feasible and clinically beneficial in patients with a variety of cancers.

Circulating tumor cells (CTCs) also contain tumor DNA and mimic tumor properties and are believed to be an alternative method of liquid biopsy (56). There are no clinical trials that tried matched therapy based on molecular alterations of CTCs currently. The reason could partly be that it is much more complex to extract DNA for sequencing after separating CTCs from the blood.

### Target Levels of Molecular Profiling

The rate of matched therapy remains low. In previous studies, although about 40% of the patients were found to have potentially actionable molecular alterations, the rate of matched therapy was less than 25% (57), indicating that only a small portion of the recruited patients had their treatment altered as a result of molecular profiling. Thus, the total benefit of genomic profiling remains unsatisfactory. Currently, most clinical trials and relevant studies rely on molecular profiling only at the DNA level without integrating RNA or protein information, which would affect the number of patients who can potentially benefit from precision medicine approaches. RNA and protein information are important for at least two reasons. First, they can be a complementary for identifying genomic alterations at the DNA level (47, 58). Second, RNAs and proteins, rather than DNAs, are the main executers of cellular behavior, and it would be more reasonable to target these molecules when they are aberrant (59). Therefore, transcriptome- and proteome-based precision medicine might not only increase the number of actionable targets, but also lead to more direct intervention in patients.

However, whether incorporating genomic and other -omic profiling could increase the match rate and further improve patients’ outcomes remain unknown. The CoppO trial (NCT02290522) enrolled 591 patients who had exhausted their treatment options, and 392 of them were found to have potentially actionable targets (41). Notably, more than half of these potentially actionable targets were revealed from RNA analysis, and the 101 patients who received matched treatment acquired a median PFS of 12 weeks, suggesting that genomic and transcriptomic profiling could be useful for personalized cancer treatment. The WINTHER trial (NCT01856296) is a multicenter study using advanced genomic and transcriptomic platforms with 303 patients consented and 107 patients treated (40). In total, 69 participants in the DNA-guided arm and 38 in the RNA-guided arm have been analyzed. A general disease control rate (DCR) of 26.2% was recorded, and the proportion of patients with a PFS2:PFS1 >1.5 was 22.4%. Intriguingly, patients in both arms had similar outcomes, indicating that RNA-guided approaches might be as useful as DNA-guided strategies. In addition, consistent with the I-PREDICT trial, a higher matching score was associated with longer PFS.

There is little evidence suggesting that targeting molecular alterations at the protein level, off-label, would improve patients’ outcome. In the MOSCATO-01 trial, nine patients with MET-positive IHC received matched therapy, and three of them had a PFS2:PFS1 >1.3, indicating that targeting MET at the protein level, off-label, is beneficial (30). However, no current trial is studying targeted therapy based on proteomic results. The Clinical Proteomic Tumor Analysis Consortium is one onco-proteogenomic effort, which aims to unravel the different proteogenomic subtypes of tumors, thus identifying driver mutations, and it will study post-translational modifications. A deeper understanding of tumor biology at the protein level will lead to proteomic analysis being incorporated into future precision trials.

### Interpretation of Molecular Profiling Results

The interpretation of molecular profiling is also challenging. Not all patients who receive matched therapies respond, which demonstrates the challenges of interpretation of the molecular profiling information and the definition of their clinical actionability. More analytical approaches and a deeper understanding of cancer biology will enable the detection of more driver mutations, and hundreds of novel targeted drugs are in clinical development, thus making the data even more complex. Mutations should be classified in a tumor type–specific manner according to the level of evidence that a mutation is a predictive biomarker for a targeted drug. Negative matched therapy should be carefully avoided, such as targeted therapy against alterations of the PI3K-AKT axis in the presence of KRAS or BRAF or another MEK pathway mutations.

The European Society for Medical Oncology (ESMO) created a framework to rank genomic alterations (60), and there are also some precision oncology knowledge databases to help physicians define targetability (61, 62). However, these resources cannot substitute for expert-guided decisions; therefore, an experienced multidisciplinary molecular tumor board, consisting of at least oncologists, pharmacologists, and bioinformatics experts, should be set up to review molecular profiling reports and provided recommendations. Given that physicians have a less stringent definition of actionability than experts in the fields of medical genetics and cancer biology (63, 64), they should also be educated to interpret genomic tests and to publish the outcomes of patients in cases of multiple targetable alterations. Unlike the classic multidisciplinary team strategy in clinical practice, there is currently no consensus on the interpretation of molecular profiling. The results of data interpretation might vary considerably depending on the interpreters’ knowledge. This field is still in its early phase and will develop rapidly with the help of artificial intelligence technology.

### Accessibility of Targeted Drugs

Current precision medicine is largely limited by the available drugs. Tumor genetic landscape testing has revealed a collection of potential targets; however, many of them do not have available drugs. It is believed that the lack of approved agents for actionable molecular alterations accounts for the unanticipated low response rate in previous studies. Moreover, performing trials within a large cancer center with more access to clinical trials could improve the possibility of matched treatment. Off-label use of commercial drugs and experimental use of preclinical chemicals could expand the treatable population and increase the response rate to treatment. For those drugs that are not approved by local administrations but have been approved in other countries, resolving the accessibility of these drugs has practical significance because these drugs probably work in patients. Currently, the use of drugs approved in one type of cancer but not in others (i.e., off-label) is probably the area in which precision medicine can do the most good. The ongoing TAPUR trial aims to fill this knowledge gap. Chemicals that are only in the stage of clinical trials, but are not approved by any administrations, might cover many targets detected using molecular profiling; however, the utility of these chemicals might be overestimated because they have not gained sufficient clinical data as proof of their efficacy. For instance, the SHIVA trial indicates that the off-label use of molecularly targeted agents did not improve the PFS of heavily pretreated patients (36).

When choosing suitable drugs or chemicals for detected meaningful targets, several issues emerge. First, one mutation might have more than one potential drug or chemicals, and one drug might work on several targets. The complicated associations between targets and drugs make it very difficult to conduct clinical trials to answer these questions. Second, more than one drugable target [e.g., germline *BRAC1* mutation with a tumor mutational burden (TMB) > 20/mega base] may be identified from one patient, and the choice of preferred target is an open question. If several driver genomic alterations coexist, a combination of targeted therapies should be considered. The combination of a BRAF inhibitor and a MEK inhibitor has been shown to be more effective than a single agent in treating melanoma (65). The I-PREDICT trial was an early test of this concept, and 18 (24.6%) enrolled patients received combination regimens, which showed that the matching score is associated with better outcomes, favoring the strategy of combination therapy. Additional studies with larger sample sizes and well-designed protocols are needed.

### Indications for Molecular Profiling

Not all patients who receive matched therapies respond. Although physicians obtain increasing amounts information concerning tumor molecular alterations, only a minority of the patients will respond to the targeted therapy. In the current model, precision medicine has been offered to patients with late-stage disease who were refractory to treatment, which reduced the likelihood that a targeted drug would be effective. Even if off-label drugs are recommended for actionable mutations, some patients may not have the chance to start the treatment because of rapid disease progression. This strategy is not optimal, suggesting that both tumor sequencing and the application of targeted therapy should be performed at earlier stages.

The rationale of precision medicine in cancer treatment is still under test; thus, most participants in clinical trials were in the late disease stage and were heavily pretreated. Some drugs with high response rates, such as Herceptin, Lynparza, and Crizotinib, could be used as first-line therapy in patients whose tumors harbor corresponding molecular alterations. However, there are many more urgent issues to be resolved before this question takes center stage.

## Conclusions

Current data concerning molecular profiling–based targeted therapy for patients with cancer demonstrates the prospect of this approach to gain clinical benefits. Though precision oncology is increasingly used in the clinical practice, more well-designed studies are urgently needed to confirm the efficacy of this strategy. Limited by the knowledge of tumor biology, precision oncology remains an investigational strategy rather than a widely used application.

## Author Contributions

ZQ and FQ contributed equally to this work. All authors contributed to the article and approved the submitted version.

## Funding

This work was funded by the National Key Research and Development Program (2019YFC1316000, 2019YFC1315802), National Natural Science Foundation of China (81871320, 81802355, 81871925), Zhejiang Distinguished Youth Foundation (LR20H160002), the Fundamental Research Funds for the Central Universities, and Medical Technology Project of Zhejiang Province (2017RC003,2020382292).

## Conflict of Interest

The authors declare that the research was conducted in the absence of any commercial or financial relationships that could be construed as a potential conflict of interest.
